# Protein Intake and Physical Activity Levels as Determinants of Sarcopenia Risk in Community-Dwelling Older Adults

**DOI:** 10.3390/nu16091380

**Published:** 2024-05-02

**Authors:** Isobel L. Stoodley, Bronwyn S. Berthon, Hayley A. Scott, Evan J. Williams, Penelope J. Baines, Hannah Knox, Sophie Wood, Beauty Paradzayi, David Cameron-Smith, Lisa G. Wood

**Affiliations:** 1 School of Biomedical Sciences and Pharmacy, University of Newcastle, Callaghan, NSW 2308, Australia; isobel.stoodley@uon.edu.au (I.L.S.); bronwyn.berthon@newcastle.edu.au (B.S.B.); hayley.scott@newcastle.edu.au (H.A.S.); evan.j.williams@newcastle.edu.au (E.J.W.); penelope.chan@newcastle.edu.au (P.J.B.); hannah.knox@newcastle.edu.au (H.K.); sophie.wood10@uon.edu.au (S.W.); beauty.paradzayi@uon.edu.au (B.P.); 2 Immune Health Research Program, Hunter Medical Research Institute, Newcastle, NSW 2305, Australia; 3 Singapore Institute of Food and Biotechnology Innovation (SIFBI), Agency for Science, Technology and Research (A*STAR), 14 Medical Drive, #07-02 MD6, Singapore 117599, Singapore; david_cameron-smith@sifbi.a-star.edu.sg

**Keywords:** sarcopenia, physical activity, protein, elderly, aging, body composition, lean mass

## Abstract

Community screening for sarcopenia is complex, with barriers including access to specialized equipment and trained staff to conduct body composition, strength and function assessment. In the current study, self-reported dietary protein intake and physical activity (PA) in adults ≥65 years was assessed relative to sarcopenia risk, as determined by body composition, strength and physical function assessments, consistent with the European Working Group on Sarcopenia in Older People (EWGSOP) definition. Of those screened (*n* = 632), 92 participants (77% female) were assessed as being at high risk of developing sarcopenia on the basis of dietary protein intake ≤1 g∙kg^−1^∙day^−1^ [0.9 (0.7–0.9) g∙kg^−1^∙day^−1^] and moderate intensity physical activity <150 min.week^−1^. A further 31 participants (65% female) were defined as being at low risk, with both protein intake [1.2 (1.1–1.5) g∙kg^−1^∙day^−1^] and PA greater than the cut-off values. High-risk participants had reduced % lean mass [53.5 (7.8)% versus 54.8 (6.1)%, *p <* 0.001] and impaired strength and physical function. Notably, high-risk females exhibited greater deficits in lean mass and strength, with minimal differences between groups for males. In community-dwelling older adults, self-reported low protein intake and low weekly PA is associated with heightened risk for sarcopenia, particularly in older women. Future research should determine whether early intervention in older adults with low protein intake and PA attenuates functional decline.

## 1. Introduction

Due to the globally aging population [1], sarcopenia (the age-related loss of muscle mass, strength and function) [2] is an increasing health concern. Sarcopenia is associated with significant morbidity and mortality [3,4,5,6] and is multifactorial [2,7,8,9]. The European Working Group on Sarcopenia in Older People (EWGSOP) [2,7] definition is widely applied for diagnosis in Western countries; however, this assessment requires access to specialised equipment (handgrip dynamometers, body composition scanners) and trained clinical personnel [10]. These barriers can hinder early identification, thereby delaying the initiation of prevention strategies or access to treatment.

One key area highlighted by the EWGSOP in recent revisions was “How can we identify older persons at high-risk of sarcopenia?” [7]. Techniques to identify individuals with pre-sarcopenia, or those with a high risk of developing sarcopenia, have had limited research. Whilst low muscle mass or poor function has been examined [4,11,12,13,14,15,16], similar to the original EWGSOP definition, this still requires access to specialised technology and trained personnel. To overcome this barrier, questionnaires have been developed, such as SARC-F, SARC-CAlF, MSRA-5 and MSRA-7, which aim to screen for sarcopenia in a community setting and prompt further assessment [17]. These questionnaires focus on physical function such as ability to climb stairs, assistance walking, recent falls or hospitalisation history [17]. Of note, only the MSRA questionnaires incorporate the causes of sarcopenia, such as poor physical activity or diet. While the future of sarcopenia assessment must include a screening tool, research to date is conflicted on the specificity and sensitivity of these currently available tools [17,18,19]. Identifying optimal strategies to diagnose sarcopenia and sarcopenia risk in community-dwelling elderly is imperative.

There are no recommended pharmaceutical treatments for sarcopenia [7], which is instead predominantly treated with lifestyle interventions such as increasing protein intake and physical activity (PA) [5,7,20,21]. Additionally, poor dietary intake (especially protein) and sedentary lifestyle are known causes of sarcopenia [8]. Therefore, inclusion of these as sarcopenia screening criteria may assist in identifying older adults at high risk of developing sarcopenia, without the need for specialised and expensive equipment. Current recommendations suggest a daily protein intake of at least 1.0 g per kilogram body weight, per day (g∙kg^−1^∙day^−1^) [8,22]. Furthermore, regular PA, including regular resistance training and 150 min moderate-intensity exercise per week (min∙week^−1^) as advised by World Health Organisation guidelines [23] and Australian Physical Activity Guidelines [24], have also been recommended by international experts to prevent and treat sarcopenia [25].

The current study therefore aimed to determine if, in community-dwelling older adults (≥65 years), identifying those who do not meet dietary protein intake and PA guidelines (methodologies that require limited specialist equipment and training) would be a suitable strategy to detect individuals with heighted sarcopenia risk, compared to those who did meet dietary protein intake and PA guidelines. For this, sarcopenia risk was determined using a comprehensive assessment with methodologies that align with sarcopenia definitions, including body compositional analysis via dual energy X-ray absorptiometry (DEXA), strength measured by dynamometry and physical function using validated assessments such as short physical performance battery (SPPB) [26] and timed up and go (TUG) [27].

## 2. Materials and Methods

### 2.1. Study Design

This study was a cross-sectional analysis of community-dwelling older adults from the Hunter and Central Coast regions of NSW, Australia. This study was conducted according to the guidelines laid down in the Declaration of Helsinki and approved by the Hunter New England Human Research Ethics Committee (2018/ETH00333, 18/08/15/4.04). All participants provided written informed consent.

### 2.2. Participants

Participants were non-smoking older adults (≥65 years) recruited between November 2018 and June 2021 from existing research volunteer databases and media releases. Participants were screened using questionnaires over the phone and, if eligible, were invited to attend a single clinic visit. Participants categorised as high risk (Section 2.3) were invited to participate in a randomized controlled trial testing the effects of a protein supplement and exercise on body composition in older people (publication in preparation). Exclusion criteria included: not meeting the risk of sarcopenia criteria (see Section 2.3), BMI > 40 kg∙m^2^, significant weight loss (>5% body weight) in the past six months, current use of systemic anti-inflammatory and/or immunosuppressant medications (e.g., corticosteroids), current use of medication that may affect muscle metabolism (e.g., thyroxine), diagnosis of renal or hepatic failure, terminal illness, or human immunodeficiency virus.

### 2.3. Risk of Sarcopenia Categorisation

Participants were categorized as being at high risk of developing sarcopenia if they had low usual protein intake (≤1.0 g∙kg^−1^∙day^−1^) and did not meet recommended PA levels (<150 min moderate-intensity exercise and/or <80 min vigorous-intensity exercise, per week) (Table 1). Low-risk participants were those with a high protein intake (>1.0 g∙kg^−1^∙day^−1^) who met recommended PA levels (Table 1). Older adults who did not meet both cut-points for a group (e.g., low protein intake but high PA levels, or vice versa) were not eligible to participate. 

The cut-point for low protein intake was derived from current recommendations, which suggest that the optimal protein level for older adults is between 1.0–1.2 g per kilogram body weight, per day (g∙kg^−1^∙day^−1^) [8,22]. The cut-point for PA levels was based on the Physical Activity Across the Life Stages report ([28]), which recommended at least 30 min of moderate-intensity exercise, five days per week for older adults. This cut-point was applied in combination with an added criteria for resistance exercise (≤1 resistance exercise session∙week^−1^), as there are no specific strength-based activity guidelines for this age group [28].

### 2.4. Body Composition

Height and weight were measured using a wall-mounted stadiometer (Seca 220; Seca, Hamburg, Germany) and electronic scales (Nuweigh EB8271; Newcastle Weighing Services, Mayfield West, Australia), both calibrated annually. Height was measured (in duplicate) to the nearest millimetre, with no shoes on. Lean muscle mass, bone mineral content (BMC) and fat mass were measured using dual energy X-ray absorptiometry (DEXA, DEXA Lunar Prodigy; GE Medical Systems, enCore 2017 software Version 16, Madison WI, USA). Regional compartments were calculated (kg) using the manufacturer’s software. Fat-free mass (FFM) was calculated by DEXA as the sum of total lean muscle mass (LMM) and BMC. Fat-free mass index (FFMI) was calculated as FFM∙height (kg/m^2^) [29,30]. Appendicular skeletal muscle mass (ASMM) was calculated as the sum of the skeletal muscle in the arms and legs [31]. Appendicular skeletal muscle mass index (ASMMI) was calculated as ASMM∙height (kg∙m^2^) [31]. Fat mass index (FMI) was calculated as fat mass∙height (kg/m^2^) [29,30]. Bone mineral density (g/cm^2^, BMD) was calculated from DEXA scans of the anteroposterior spine (AP) and femur. AP spine BMD was calculated from L1–L4 vertebrae. When vertebrae had significant osteoarthritis, or surgical implants that might affect BMD results, these vertebrae were excluded from analysis. Osteopenia and osteoporosis were defined as recommended by the World Health Organisation; a *T*-score between −1.0 and −2.5 was defined as osteopenia and a *T*-score of 2.5 or lower was defined as osteoporosis [32]. Quality assurance and quality control measures were performed daily per the manufacturer’s instructions [33].

### 2.5. Strength and Physical Function

Short physical performance battery (SPPB), five chair stand test (time taken to complete five stands), thirty second sit-to-stand (30STS, how many stands completed in 30 s) and timed up-and-go (TUG, time taken to stand from a chair, walk 3 m, turn around and sit back down) were used assess balance, functional mobility and lower limb strength. SPPB includes three tests, each assessed out of four points: holding side-by-side, semi-tandem and tandem position for ten seconds; the five chair stand test as described above; and a 4 m walk test. The total score is reported out of 12 and gait speed from the 4 m walk test was reported separately from total SPPB score. Handgrip strength (kg) was measured using a Jamar handgrip dynamometer (JAMAR 5030J1 Hand Dynamometer, Performance Health ANZ, North Ryde, NSW, Australia). Shoulder adduction and abduction (kg) were measured (TTM Shoulder and Arm Dynamometer).

### 2.6. Questionnaires

#### 2.6.1. Dietary Intake

A minimum of three 24-h food recalls (including one weekend day) were used to assess dietary intake and usual protein intake for sarcopenia risk categorisation. Nutrient intake was quantified by a dietitian using FoodWorks (Professional Edition Version 8.9; Xyris Software Pty Ltd., Kenmore Hills, Queensland, Australia) [34]. Usual protein intake was calculated as described in Section 2.3. Under-reporting was assessed using the Goldberg method [35,36] and used to compare between groups.

#### 2.6.2. Physical Activity

During telephone screening, participants were asked to describe their usual PA patterns, including types, duration and frequency of activities, over the past three months. PA was graded by intensity under supervision of the study physiotherapists (P.C. and H.K.) and summed to calculate weekly PA time. Time spent doing moderate or vigorous activity and resistance training sessions were used to assess risk of sarcopenia (Section 2.3). To further quantify physical, work, home-based and recreational activity, participants completed the validated Yale Physical Activity Questionnaire (YPAS) [37].

#### 2.6.3. General Health and Health Related Quality of Life

Participants were asked to recall their medical history, smoking history, pack years, current medications and supplements. The Research and Development Corporation (RAND) 36-item Health Survey (SF-36) was used to measure health-related quality of life [38]. Summary scores (physical component score and mental component scores) were calculated using algorithms designed by the SF-36 developers, using Australian population means [39,40].

### 2.7. Sarcopenia Assessment

Sarcopenia was defined according to the operational definition revised by the 2019 European Working Group on Sarcopenia in Older People (EWGSOP) [7]. Probable sarcopenia was defined as low muscle strength (grip strength < 27 kg for males, <16 kg for females, or five chair stand test > 15 s). Sarcopenia was defined by low muscle mass [ASMMI < 7.0 kg/m^2^ for males or <6.0 kg/m^2^ for females, as determined by DEXA] in addition to meeting low muscle strength cut-points. Additionally, if poor physical performance (SPPB ≤ 8, gait speed ≤ 0.8 m∙s^−1^ and/or TUG ≥ 20 s) was identified, sarcopenia was defined as severe. The functional cut-points from the EWGSOP definition were evaluated independently of the low muscle and low strength prerequisites to assess physical function impairment.

### 2.8. Statistical Analysis

Data were analysed using Stata 15.1 (Stata Corporation, College Station, TX, USA). Normality was assessed using Shapiro–Wilk normality testing. Data are reported as mean (standard deviation, *SD*) for normally distributed data or median (interquartile range, IQR) for non-normally distributed data. Baseline data were compared between groups using unpaired Student’s *t*-test or Mann–Whitney U test. Categorical variables were analysed using the chi-squared test or Fisher’s exact test. Dietary data were analysed using the residual method to adjust for energy intake, as described by Willett et al. [41]. One-way ANOVA (normally distributed data) and the Kruskal–Wallis test (non-normally distributed data) with post hoc testing (using adjusted *p*-values) were used to compare sex differences between the high- and low-risk groups. Pearson’s correlation or Spearman’s rank correlation were used to assess associations. Risk factors such as usual protein intake, total weekly exercise time, age, sex and lean muscle mass were tested in multiple linear regression analysis. Risk factors were grouped in relevant models and the robust variance estimator was applied to all variables. Variance of residuals and multivariate normality were assessed in each model. *p*-values of <0.05 were considered statistically significant. Graphs were produced using GraphPad Prism 10.0 (GraphPad Software, La Jolla, CA, USA).

#### Sample Size Calculation

Based on previous studies [42], to have 80% power to detect a mean difference in FFM of 0.6 (*SD* = 0.9) kg, with two experimental subjects per control subject, we needed to study 54 experimental subjects and 27 control subjects to be able to reject the null hypothesis that the population means of the experimental and control groups are equal. The Type I error probability associated with this test of this null hypothesis was 0.05.

## 3. Results

### 3.1. Baseline Characteristics

Participant recruitment began in November 2018 and was completed June 2021. A total of 632 participants were screened (Supplementary Figure S1), and in total, 92 participants met the low PA and low protein intake requirement for the “high risk of developing sarcopenia” group and 31 participants with high PA levels and high protein intake in the “low risk of developing sarcopenia” group. Participant characteristics are described in Table 2. Participants from both groups were similar for age, sex and smoking history. There were significantly more participants with hypertension (*p* = 0.008) and using cholesterol-lowering medication (*p* = 0.032) in the high-risk group.

### 3.2. Body Composition, Strength and Physical Function by Group and Sex

**Figure 1:**
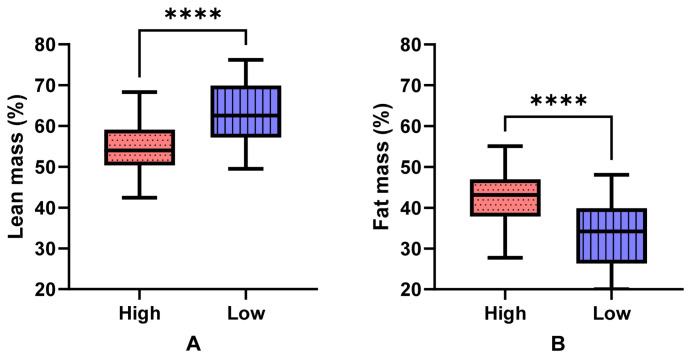
Differences in (**A**) lean muscle percentage and (**B**) fat percentage by risk status. ****, *p* < 0.0001. Median, interquartile range, min and max presented.

Table 3 describes the differences in body composition, strength and function between older adults at high risk and low risk of developing sarcopenia and then further subdivided by sex. The high-risk group had a significantly lower percentage of lean mass compared to the low-risk group [53.5 (7.8)% versus 54.8 (6.1)%, *p <* 0.001] (Figure 1A). The high-risk group had a significantly higher weight and BMI than the low-risk group (*p <* 0.001), with higher fat mass and FMI (*p <* 0.001) (Table 3, Figure 1B). The high-risk group had poorer upper and lower limb strength and physical function (Table 3).

**Figure 2:**
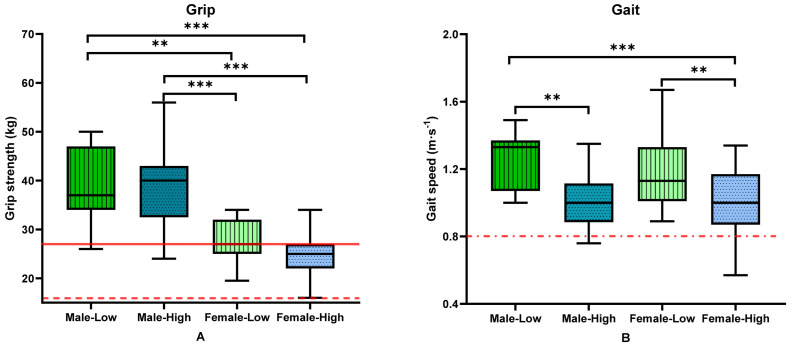
Differences in clinical measures by sex and risk status in (**A**) handgrip strength, (**B**) gait speed. Data analysed using ANOVA or Kruskal–Wallis. ***, *p* < 0.001; **, *p* < 0.01. Red lines for EWGSOP cut-points, solid line for males, dotted for female and solid/dot alternate for combined cut-point. Median and interquartile range presented.

Body composition, strength and function were further subdivided by risk group and sex (Table 3). There was little difference in outcomes between the male groups, with both male groups having higher muscle mass, BMC and strength than the female groups (Table 3). VAT mass was higher in the male high-risk group compared to the female high-risk group and both low-risk groups (*p <* 0.001). The females categorised as high-risk generally had the poorest body composition, strength and function, indicated by a lower muscle mass (*p <* 0.001), higher fat mass (and percentage fat mass) (*p <* 0.001) and poorer leg strength, shoulder adduction strength and shoulder abduction strength (*p <* 0.001). The high-risk groups had the poorest function outcomes, with poorer grip strength (Figure 2A), gait speed (Figure 2B) and TUG time in males and females categorised as high-risk compared to the low-risk groups.

### 3.3. Sarcopenia Diagnosis according to the EWGSOP Definition of Sarcopenia

Two participants met the EWGSOP criteria for sarcopenia or severe sarcopenia: one in each group (Table 3). There was a trend for the proportion of participants in the high-risk group who met the EWGSOP criteria for probable sarcopenia to be higher than the low-risk group (15% versus 3%, *p =* 0.087). When considering only the EWGSOP functional cut-points for severe sarcopenia (independent of muscle mass and strength), 17 participants in the high-risk group met these criteria compared with no participants in the low-risk group (*p =* 0.012). Of these participants, *n =* 14 were high-risk females, compared to *n =* 3 high-risk males (20% versus 14%, *p =* 0.071).

Participants meeting functional cut-points for sarcopenia were compared to the other high-risk participants in key outcomes (Supplementary Tables S1–S4). There were no significant differences in dietary macronutrient intake or physical activity levels between the two groups. Participants meeting functional cut-points had impaired strength and function outcomes compared to other high-risk participants, as well as poorer quality of life in the domains of physical function, role limitations due to physical function, general health, energy and overall physical component score. Participants meeting functional cut-points compared to other high-risk participants were more likely to have a history of hernia (18% versus 3%, *p =* 0.042) and require mobility aids (18% versus 0%, *p =* 0.005), with no other differences in self-reported medical history.

### 3.4. Dietary Intake

Recent dietary intake differed in high-risk and low-risk groups (Table 4). High-risk participants had a lower intake of dietary energy, protein, total fat, monounsaturated fatty acids, polyunsaturated fatty acids and fibre (*p* < 0.001). When nutrient intakes were adjusted for total energy intake, differences in protein, carbohydrate and saturated fatty acids, remained significant between the groups. Under-reporting was prevalent in the high-risk group, with 30% of high-risk participants categorised as under-reporters, which was significantly higher than the low-risk group with no under-reporters by the Goldberg method (*p* = 0.001).

### 3.5. Physical Activity

PA levels, as measured by the YPAS, are described in Table 5. The high-risk group had significantly lower vigorous activity units (*p <* 0.001), moving units (*p =* 0.001) and higher sitting units (*p =* 0.002) per month compared to the low-risk group. This led to overall lower total activity dimension indices (*p <* 0.001) and total activity time (high risk 25.3 (16.8–37.3) versus low risk 32.3 (24.3–38.8), *p =* 0.037) in the high-risk group. When comparing exercise activities, the high-risk group spent less time completing: brisk walking (*p <* 0.001), stretch or yoga style activities (*p =* 0.032), aerobic style activities (*p <* 0.001) and strength exercise (*p <* 0.001) compared to the low-risk group.

### 3.6. Quality of Life

There were significant differences in quality of life between high-risk and low-risk participants (Supplementary Table S5). Participants at high risk of sarcopenia had more physical limitations (*p <* 0.001), lower general health scores (*p =* 0.006) and lower energy scores (*p =* 0.011). There were no significant differences between participants in emotional health sub-domains or the overall mental component score.

### 3.7. Relationship between Determinants and Clinical Sarcopenia Outcomes

Correlations between selected risk factors and clinical outcomes of sarcopenia are described in Table 6. There were weak–moderate correlations between upper body strength and muscle mass (both FFMI and ASMMI), while FMI was inversely correlated with 30STS (r_s_ = −0.424, *p <* 0.001), grip strength (r_s_ = −0.380, *p <* 0.01), gait speed (r_s_ = −0.428, *p <* 0.001) and TUG time (r_s_ = 0.330). Protein intake was moderately inversely correlated with FMI (r_s_ = −0.577, *p <* 0.001) and weakly correlated with strength and physical function: 30STS (r_s_ = 0.299, *p <* 0.05), gait speed (r_s_ = 0.266, *p <* 0.05) and TUG (r_s_ = −0.298, *p <* 0.05). Energy intake was strongly correlated with protein intake (r_s_ = −0.758, *p <* 0.001) and weakly correlated with muscle mass indices, strength and physical function outcomes. Interestingly, energy was inversely correlated with FMI (r_s_ = −0.302, *p <* 0.001). Total exercise time was weakly correlated with gait speed (r_s_ = 0.321, *p <* 0.01). Strength training time was weakly correlated with FMI (r_s_ = −0.393, *p <* 0.001), lower limb strength (both five chair stand test and 30STS), upper body strength (grip strength and shoulder abduction strength) and function (gait speed and TUG). Aerobics time was weakly correlated with FMI (r_s_ = 0.292, *p <* 0.05) and shoulder abduction (r_s_ = 0.227, *p <* 0.05).

Multiple linear regressions were used to investigate associated factors of body composition, strength and function (Table 7 and Table 8). Protein intake had a positive association with gait speed; this effect was maintained after adjustment for total exercise time, age, sex and muscle mass. Both usual protein intake (g∙kg^−1^∙day^−1^) and weekly exercise time (hours∙week^−1^) were associated with better TUG time; however after adjusting for other variables, only exercise time and age were significant. Grip strength was associated with age (poorer), male sex and muscle mass (better); however, it was not associated with protein intake or exercise. Shoulder adduction strength was positively associated with exercise time but only in Model 2 and 3, which adjusted for age, sex and muscle mass (Model 3). Protein intake was negatively associated with both spine and femur BMD (Table 8). Male sex was associated with better BMD, greater muscle and lower fat mass. Protein intake and exercise time were significant variables associated with both lean muscle mass and fat mass; this model was strengthened when sex was accounted for in the model.

## 4. Discussion

This study aimed to compare clinical markers of sarcopenia in older adults with low usual protein intake (≤1 g∙kg^−1^∙day^−1^) and low PA levels (≤150 min∙week^−1^) (high-risk) to older adults with moderate–high protein intake who met recommended PA levels (low-risk). High-risk adults had lower percentage lean mass, higher fat mass and poorer strength and function. High-risk participants also reported greater physical limitations and overall poorer physical health than the low-risk group. These differences were more pronounced in the female participants, with high-risk females displaying poorer body composition, strength and function compared to low-risk females and both male groups. There were fewer differences between the male groups, indicating that dietary protein intake and PA as screening criteria may be more relevant for females. As significant differences in body composition, strength, physical function and quality of life were observed, using ≤1 g∙kg^−1^∙day^−1^ protein intake and ≤150 min∙week^−1^ moderate PA appeared beneficial as screening tools for identifying older adults with impaired function, who may be at high risk of developing sarcopenia.

Protein intake and PA levels were the key risk factors for sarcopenia that were utilized in this study. After adjusting for potential confounders, such as age, sex and muscle mass, protein and/or exercise levels were still associated with faster gait speeds, quicker TUG time, greater shoulder adduction strength, increased lean muscle and decreased fat mass. Consistent with our results, in a pooled analysis of four longitudinal cohorts, Mendonça et al. [43] found that higher protein intakes (≥0.8 g∙kg^−1^∙day^−1^) were associated with faster gait speed, reduced functional decline over time and the development of fewer mobility limitations, after adjusting for sex, age and education. There were limited interactions between protein and PA levels, with similar positive associations between protein and gait speed within each low, medium and high PA category [43]. Another recent study utilizing similar cut-points to this study (≤1.1 g∙kg^−1^∙day^−1^ protein and the same cut-points for PA) found high protein and high PA were associated with increased chair stands, gait speed, SPPB score and lower fat mass [44]. Interestingly, PA levels alone were not associated with muscle mass, while the PA and protein intake interaction had the strongest associations for strength and function variables. Our study confirmed these findings, while also expanding to report poorer quality of life in participants with below recommended physical activity and protein intake. It is important to note that in our study, the high-risk group was very sedentary with nil median exercise activities. A larger sample size may have increased the range of exercise time in the high-risk group to further elucidate correlations with sarcopenia-related outcomes. Our results in this study indicate that sedentary lifestyles, with no exercise activities during the week, increases the risk of poor physical function and may contribute to the development of sarcopenia. As current sarcopenia definitions do not incorporate physical function until diagnosing “severe” sarcopenia, it is crucial to develop screening tools that can identify individuals prior to functional decline. This study, in addition to recent research [43,44,45], highlights the growing importance of protein intake and PA not only as treatment modalities, but as screening tools for future sarcopenia and functional decline.

In addition to muscle mass differences, the high-risk group also had higher fat mass (kg, index and percentage). Interestingly, the high-risk group also reported lower energy intake (likely confounded by the larger proportion of under-reporters in this group), while energy intake overall was correlated positively with muscle mass, strength and function outcomes and inversely correlated with FMI. Adiposity increases with age [46,47] and, while adipose tissue was previously thought inert, it is now known to produce and release pro-inflammatory agents such as adipokines [48]. The higher fat mass identified in our high-risk participants, which was correlated with poorer leg strength, grip strength, gait speed and TUG time, may have contributed to the differences in strength and physical function observed, by augmenting systemic inflammation. This was similar in other cohorts, for example in a Taiwanese cohort where body fat indices were negatively associated with gait speed and grip strength [49]. In another study of nursing home residents, a higher body fat percentage was associated with poorer five chair stand test time and handgrip strength [50]. In a New Zealand cohort, grip strength was significantly associated with ASMMI; however, this association was not significant in obese participants [51]. A meta-analysis of 50 studies found that high BMI (≥30) and low muscle strength were independent predictors of functional decline, while low muscle mass was not [52]. This suggests that adipose tissue could play a confounding role in muscle strength and function and would be important to consider in the definition and diagnosis of sarcopenia. However, given the low-risk groups reported a higher energy intake, total dietary intake may be limited as a surrogate measure for identifying individuals with poor body composition and risk of sarcopenia.

Sex differences were also identified in this analysis. We found that males, regardless of protein intake or exercise levels, had similar outcomes except for gait speed and TUG time, which were poorer in the high-risk group. When comparing high- and low-risk females, higher protein intake and activity levels were associated with better leg strength (five chair stand test and 30STS), higher SPPB score, faster gait speed and TUG time. In fact, most participants who met the impaired functional EWGSOP criteria were female. Interestingly, poor nutritional intake and physical inactivity have been recently identified as predictive risk factors for sarcopenia development for females, but not for males, in a Korean cohort study [53], which corroborates these sex-dependent effects observed. While it is well known there are sex differences in aging and skeletal muscle health, these effects are not completely understood [54]. While males generally have more muscle mass than females, both sexes experience a decline in sex hormones during aging; testosterone declines between 2–3% in males each year after 30 years of age [55], while estrogen declines in females post-menopause [56]. These hormonal changes may mediate declines in muscle health, as testosterone is known to activate mammalian target of rapamycin pathways contributing to skeletal muscle hypotrophy [57], while estrogen appears to have beneficial effects on muscle recovery, potentially by reducing inflammation [58]. Higher baseline levels of muscle mass and potentially smaller declines in sex hormones may explain the differences between males and females in this cohort. However, without analysing sex hormone levels, this would be hard to elucidate. Nevertheless, this work suggests that classifying sarcopenia risk according to protein intake and PA level may be more relevant in females than males, as females had significant differences in strength and function results dependent on group categorisation. This would be worth exploring further in larger cohorts, as different cut-points may be required to identify poorer function in older males.

Our screening criteria of low protein intake and low physical activity did not identify individuals with sarcopenia. Two participants, one in each group, met the EWGSOP criteria for sarcopenia or severe sarcopenia; however, there was a slightly higher proportion of individuals with “probable sarcopenia” in the high-risk group. When using the functional criteria of severe sarcopenia, independent of muscle mass categorisation, 19% of the high-risk group met these criteria, compared to 0% in the low-risk group and this was reflected with poorer quality of life scores in physical function-related domains. While sarcopenia has historically been defined by the presence of low muscle mass, newer definitions are moving away from muscle mass as a key characteristic. Dynapenia describes the age-related loss of muscle strength, and this is becoming more of a focus. For example, the Sarcopenia Definitions and Outcomes Consortium (SDOC) uses strength, measured by handgrip strength and gait speed, as a main distinguishing factor in the diagnosis of sarcopenia [59]. This new recommendation is partly due to the stronger predictive capacity between strength and physical function (grip strength and gait speed) and adverse clinical outcomes, compared to muscle mass [59,60]. Our results indicate that screening using dietary protein and physical activity levels presents a unique opportunity to identify individuals prior to the muscle loss currently used to define sarcopenia and prevent further declines in strength and physical function and preserve independence. Adapting protein and PA questionnaires for rapid assessment in the community, potentially even without requiring clinician administration (for example, the ASA24 [61] and Australian Healthy Eating Survey [62] can be self-administered online) would be of benefit to increase screening and self-screening in older adults who are at risk of sarcopenia.

This study suggests that lifestyle-based criteria may be valuable for identifying individuals at high risk of developing sarcopenia. It is crucial that health professionals identify individuals with poor function as early as possible, as they would benefit most from early intervention. It also highlights the need for population-based muscle strength and function reference ranges for dynapenia and pre-sarcopenia, particularly in physical function outcomes such as gait speed, TUG and grip strength. TUG, in this study for example, was not reported below the EWGSOP cut-point of 20 s for any participant; however, significant differences between high- and low-risk participants were observed. As other studies and meta-analysis in community-dwelling older adults have observed similar times [63,64], which are significantly quicker compared to adults in residential aged care [65], it emphasizes that updated cut-points are needed. These could be utilized in general practitioner and allied health practices, in combination with low protein and low PA screening questionnaires, to identify individuals who would benefit most from preventative interventions for sarcopenia.

### Limitations

A limitation of this cross-sectional study was its sample size and exploratory nature. A larger sample size and inclusion of participants who met either low protein intake or low PA criteria (but not both) would allow us to investigate whether protein intake or PA is more important for identifying pre-sarcopenia.

Comparing the effectiveness of our criteria to other definitions of sarcopenia (such as SDOC) would have been useful, given pre-sarcopenia or sarcopenia risk is a poorly researched area with no consensus on definition. Our study population was aged between 65–86 years old, predominantly Caucasian and all community-dwelling, so the application of this criteria in other populations such ≥85 years old; Indigenous, Asian and African demographics; and individuals in aged care residences may be limited and would require further research to confirm. Similarly, our criteria would need to be tested in a younger population (<65 years old). While traditionally not a target population for sarcopenia research, younger adults meeting our protein and activity criteria may still be at risk of developing sarcopenia and in fact identifying individuals at risk from an earlier age may be more beneficial.

Our criteria were determined from self-reported data, for both dietary intake (24-h food recall) and PA, which have known errors, although they do have a low participant burden [66,67,68]. However, the questionnaires utilized could be easily translated to a variety of settings external to research, increasing its relevance and application for community guidelines.

## 5. Conclusions

This study found that low usual protein intake (≤1 g∙kg^−1^∙day^−1^) and low PA levels (≤150 min∙week^−1^) can be used to identify older adults with lower percentage muscle mass, higher fat mass and poorer strength and physical function. Using protein and PA levels as screening tools for sarcopenia development warrants further investigation, especially in females where body composition, strength and physical function deficits were more pronounced. This study highlights the need for further research in those at a high risk of developing sarcopenia and/or pre-sarcopenia, as individuals with poorer physical performance can be identified prior to suffering significant muscle loss. These individuals would benefit from early lifestyle intervention, such as increasing protein intake and PA.

## 6. Clinical Significance

- A screening tool assessing lifestyle-based criteria, which does not require access to specialist equipment and training, may assist in identifying older adults at risk of sarcopenia.
- Older adults who are active and eat well appear to be at lower risk of developing sarcopenia.
- Older adults who are sedentary and consume low-protein diets may be at higher risk of developing sarcopenia, with higher risk in females than males.
- Assessing and improving the activity levels and protein intake, of older adults prior to sarcopenia diagnosis may prevent functional decline linked with sarcopenia
- Individuals with early functional decline might not meet current sarcopenia definitions, but may already have impaired body composition, strength and physical function. Community-dwelling older adults should engage with allied health professionals such as dietitians, physiotherapists and exercise physiologists early for nutrition and physical activity-based sarcopenia prevention strategies.

## Figures and Tables

**Table 1:** Summary of group criteria for usual protein intake and physical activity levels.

Group	Usual Protein Intake	Usual Physical Activity Levels
High risk of developing sarcopenia	≤1.0 g∙kg^−1^∙day^−1^	<150 moderate intensity and/or <80 vigorous intensity min∙week^−1^ ≤1 resistance exercise session∙week^−1^
Low risk of developing sarcopenia	>1.0 g∙kg^−1^∙day^−1^	≥150 moderate intensity and/or ≥80 intensity min∙week^−1^ >1 resistance exercise session∙week^−1^

**Table 2:** Participant demographics and health conditions.

	High Risk (*n* = 92)	Low Risk (*n* = 31)	*p*-Values
Age	72.3 (68.7–75.9)	70.3 (67.1–74.2)	0.062
Sex, female, *n* (%)	71 (77%)	20 (65%)	0.165
Ex-smokers, *n* (%)	44 (48%)	13 (42%)	0.569
Pack years	6.4 (1.3–19)	2 (0.45–16.5)	0.270
Health Conditions			
Hypertension, *n* (%)	55 (60%)	10 (32%)	**0.008**
Arthritis, *n* (%)	55 (60%)	18 (58%)	0.866
Joint replacement, *n* (%) Knee, *n* (%) Hip, *n* (%)	11 (12%) 4 (4%)	2 (6%) 1 (3%)	0.514 1.000
Spine BMD *T*-scores, *n* (%) Normal (≥−1.0) Osteopenia (−1.0 to −2.5) Osteoporosis (≤2.5)	48 (52%) 39 (42%) 5 (5%)	16 (52%) 12 (39%) 3 (10%)	0.679
Femur BMD *T*-scores, *n* (%) Normal (≥−1.0) Osteopenia (−1.0 to −2.5) Osteoporosis (≤2.5)	67 (73%) 20 (22%) 5 (5%)	17 (55%) 12 (39%) 2 (7%)	0.138
Diabetes, *n* (%)	9 (10%)	1 (3%)	0.449
Prediabetes, *n* (%)	3 (3%)	1 (3%)	1.000
Anxiety, *n* (%)	10 (11%)	5 (16%)	0.526
Depression, *n* (%)	7 (8%)	1 (3%)	0.678
Medications and supplements			
Reflux, *n* (%)	22 (24%)	7 (23%)	0.880
Cholesterol-lowering, *n* (%)	44 (48%)	8 (26%)	**0.032**
Any supplement, *n* (%)	58 (63%)	21 (68%)	0.637

Bold indicates significant difference.

**Table 3:** Body composition, strength, physical function and EWGSOP categorisation, by sex and risk status.

	Total		*p*-Values	Male		Female		*p*-Values
	High Risk (*n* = 92)	Low Risk (*n* = 31)		Low Risk (*n* = 11)	High Risk (*n* = 21)	Low Risk (*n* = 20)	High Risk (*n* = 71)	
Body Composition								
BMI (kg/m^2^)	29.6 (26.8–34.3)	25.0 (23.2–27.4)	**<0.001**	25.04 (22.62–26.83)	30.05 (27.15–33.33)	25.07 (23.775–27.865)	29.17 (26.56–34.28)	**<0.001 ^ACF^**
FFM (kg)	44.52 (41.52–51.32)	41.85 (40.22–58.59)	0.450	59.55 (55.27–61.03)	58.83 (55.41–65.23)	40.48(39.54–41.77)	42.56(40.80–46.33)	**<0.001 ^BCDE^**
FFMI (kg/m^2^)	16.89 (15.89–18.84)	16.13 (15.89–18.18)	0.221	18.44 (1.07)	19.76 (1.92)	15.93 (1.19)	16.73 (1.57)	**<0.001 ^BCDE^**
ASMM (kg)	18.66 (17.02–22.11)	17.26 (16.44–23.52)	0.461	25.63 (23.04–26.64)	25.13 (23.75–27.68)	16.60 (15.48–17.21)	17.76 (16.48–19.31)	**<0.001 ^BCDE^**
ASMMI (kg/m^2^)	7.10 (6.51–8.07)	6.71 (6.28–7.81)	0.269	7.89 (0.58)	8.46 (1.00)	6.51 (0.69)	6.96 (0.79)	**<0.001 ^BCDE^**
Fat mass (kg)	33.86 (27.28–41.31)	23.75 (18.67–27.40)	**<0.001**	19.94 (5.66)	32.80 (9.53)	25.29 (7.05)	35.57 (9.24)	**<0.001 ^ACF^**
Fat mass index (kg/m^2^)	12.43 (10.45–16.04)	8.27 (6.76–10.74)	**<0.001**	6.28 (1.65)	10.84 (3.11)	10.02 (2.91)	13.71 (3.53)	**<0.001 ^ABCEF^**
VAT mass (kg)	1.34 (0.96–1.81)	0.60 (0.28–1.20)	**<0.001**	1.05 (0.62–1.51)	2.13 (1.53–2.88)	0.48 (0.21–0.85)	1.17 (0.87–1.51)	**<0.001 ^ADEF^**
Total body bone mineral content (kg)	2.26 (2.05–2.69)	2.09 (1.90–2.96)	0.333	3.21 (2.95–3.27)	3.17 (2.97–3.30)	1.93 (1.81–2.08)	2.16 (2.03–2.40)	**<0.001 ^BCDE^**
Strength and Physical Function								
Five chair stand test (s)	11.00 (9.49–14.00)	8.68 (8.00–10.4)	**<0.001**	9 (8.57–10.00)	9.86 (9.06–13.08)	8.49 (7.38–10.46)	11.06 (10–14.00)	**<0.001 ^CF^**
Thirty second sit-to-stand (stands)	12.67 (2.90)	17.47 (3.75)	**<0.001**	16 (15–19)	13 (13–16)	17 (16–19)	12 (10–14)	**<0.001 ^CDF^**
Grip strength, total (kg)	26.0 (23.0–30.0)	29.0 (26.0–34.0)	**0.012**	37 (34–47)	40 (34.00–42.00)	27.00 (25.00–32.00)	25.00 (22.00–27.00)	**<0.001 ^BCDE^**
Shoulder adduction strength (kg)	11.5 (7.5–15.0)	16.75 (13.0–22.5)	**0.001**	23.00 (16.50–33.50)	21.50 (13.50–24.00)	13.50 (10.0–18.0)	10.0 (7.0–13.0)	**<0.001 ^BCE^**
Shoulder abduction strength (kg)	6.8 (3.5–10.8)	10.0 (8.0–15.0)	**0.002**	14.00 (11.50–16.00)	13.00 (8.5–19.00)	9.00 (6.50–10.00)	5.50 (3.0–8.5)	**<0.001 ^CE^**
SPPB score (total) <10/12 *n* (%) ≥10/12 *n* (%)	11 (10–12) 17 (18.5%) 75 (81.5%)	12 (12–12) 0 (0%) 30 (100%)	**0.001** **0.012**	12 (12–12)	12 (9–12)	12 (12–12)	11 (10–12)	**0.003 ^F^**
Gait speed (m∙s^−1^)	1.00 (0.87–1.15)	1.15 (1.03–1.33)	**<0.001**	1.33 (1.07–1.37)	1.00 (0.89–1.08)	1.13 (1.01–1.33)	1.00 (0.87–1.17)	**<0.001 ^ACF^**
TUG (s)	7.00 (6.70–7.91)	5.80 (5.00–6.18)	**<0.001**	5.53 (5.00–6.83)	7.00 (6.49–7.49)	5.87 (5.01–6.18)	7.00 (6.75–7.97)	**<0.001 ^ACDF^**
EWGSOP Sarcopenia Categorisation								
No sarcopenia, *n* (%) Probable sarcopenia, *n* (%) Sarcopenia, *n* (%) Severe sarcopenia, *n* (%)	77 (84%) 14 (15%) 0 (0%) 1 (1%)	29 (94%) 1 (3%) 1 (3%) 0 (0%)	0.087	10 (91%) 0 (0%) 1 (9%) 0 (0%)	15 (71%) 5 (24%) 0 (0%) 1 (5%)	19 (95%) 1 (5%) 0 (0%) 0 (0%)	62 (87%) 9 (13%) 0 (0%) 0 (0%)	**0.048**
Functional criteria only, *n* (%)	17 (19%)	0 (0%)	**0.012**	0 (0%)	3 (14%)	0 (0%)	14 (20%)	0.071

Data presented as median (IQR) or mean (*SD*). Bold indicates significant difference. BMI, body mass index; s, seconds; m, metres; kg, kilograms; FFM, fat-free mass; FFMI, fat-free mass index; ASMM, appendicular skeletal muscle mass; ASMMI, appendicular skeletal muscle mass index; VAT, visceral adipose tissue; SPPB, Short Physical Performance Battery; TUG, timed up-and-go test; EWGSOP, European Working Group on Sarcopenia in Older People. ^A^, significant post hoc differences between low-risk males versus high-risk males; ^B^, significant post hoc differences between low-risk males and low-risk females; ^C^, significant post hoc differences between low-risk males and high-risk females; ^D^, significant post hoc differences between high-risk males and low-risk females; ^E^, significant post hoc differences between high-risk males and high-risk females; ^F^, significant post hoc differences between low-risk females and high-risk females.

**Table 4:** Macronutrient intake by risk status.

	High Risk (*n* = 92)	Low Risk (*n* = 31)	*p*-Values	Energy-Adjusted *p*-Values
Macronutrients				
Energy (kJ∙day^−1^)	5915 (5272–7213)	7913 (6566–8707)	**<0.001**	
Energy (kJ∙kg^−1^∙day^−1^)	76.9 (63.1–76.9)	107.6 (94.5–107.6)	**<0.001**	
Protein (g∙day^−1^)	64.7 (12.8)	92.0 (15.8)	**<0.001**	**<0.001**
Protein (g∙kg^−1^∙day^−1^)	0.9 (0.7–0.9)	1.2 (1.1–1.5)	**<0.001**	**<0.001**
Carbohydrate (g∙day^−1^)	148.4 (126.6–184.3)	158.6 (131.9–246.2)	0.057	**0.007**
Fibre (g∙day^−1^)	19.3 (15.3–25.8)	26.8 (22.8–36.0)	**<0.001**	0.077
Fat (g∙day^−1^)	57.3 (18.2)	76.9 (20.5)	**<0.001**	0.675
SFA (g∙day^−1^)	22.0 (8.3)	24.9 (7.5)	0.086	**0.018**
MUFA (g∙day^−1^)	20.4 (15.9–26.5)	29.0 (24.1–36.6)	**<0.001**	0.054
PUFA (g∙day^−1^)	8.0 (6.4–9.9)	11.5 (8.8–18.5)	**<0.001**	0.126
Under-reporters				
*n*, (%)	27 (30%)	0 (0%)	**0.001**	

Data presented as median (IQR) or mean (*SD*). Bold indicates significant difference.

**Table 5:** Yale Physical Activity Survey physical activity levels by group.

	High Risk (*n* = 92)	Low Risk (*n* = 31)	*p*-Values
Activity Dimension Indices			
Vigorous Activity index (units∙month^−1^)	5.0 (0.0–10.0)	30.0 (20.0–40.0)	**<0.001**
Leisure walking index (units∙month^−1^)	16.0 (8.0–24.0)	16.0 (8.0–16.0)	0.995
Moving index (units∙month^−1^)	9.0 (6.0–9.0)	9.0 (9.0–12.0)	**0.001**
Standing index (units∙month^−1^)	4.0 (2.0–4.0)	4.0 (2.0–4.0)	0.860
Sitting index (units∙month^−1^)	2.0 (2.0–3.0)	2.0 (1.0–2.0)	**0.002**
Total activity dimension indices	36.0 (23.5–47.5)	52.0 (47.0–69.0)	**<0.001**
Activities			
Brisk walking (hours∙week^−1^)	0.0 (0.0–1.8)	1.7 (1.0–3.0)	**<0.001**
Stretch/yoga/tai chi (hours∙week^−1^)	0.0 (0.0–1.0)	0.8 (0.0–1.5)	**0.032**
Aerobics (hours∙week^−1^)	0.0 (0.0–0.0)	1.0 (0.0–1.8)	**<0.001**
Cycling (hours∙week^−1^)	0.0 (0.0–0.0)	0.0 (0.0–0.0)	0.901
Lap swimming (hours∙week^−1^)	0.0 (0.0–0.0)	0.0 (0.0–0.0)	**0.045**
Strength exercise (hours∙week^−1^)	0.0 (0.0–0.0)	1.5 (1.0–2.0)	**<0.001**
Leisurely walking (hours∙week^−1^)	0.0 (0.0–1.0)	0.0 (0.0–0.0)	0.213

Data presented as median (IQR) or mean (*SD*). Bold indicates significant difference.

**Table 6:** Correlation matrix describing relationship between body composition, strength, physical function and risk factors.

	FFMI	ASMMI	FMI	Five Chair Stand	30STS	Grip	Shoulder Adduction	Shoulder Abduction	Gait Speed	TUG	Protein Intake	Energy Intake	Exercise Time	Strength Time	Aerobics Time
FFMI	1														
ASMMI	0.946	1												Key	
FMI	0.163	0.205	1											*p* > 0.05	
Five chair stand	−0.093	−0.091	0.230 *	1										*p* ≤ 0.05	
30STS	0.092	0.081	−0.424	−0.761	1									*p* ≤ 0.01	
Grip strength	0.368	0.435	−0.380	−0.352	0.352	1								*p* ≤ 0.001	
Shoulder adduction	0.437	0.463	−0.190	−0.326	0.333	0.577	1								
Shoulder abduction	0.336	0.377	−0.231	−0.149	0.154	0.519	0.658	1							
Gait speed	−0.118	−0.042	−0.428	−0.401	0.45	0.266 *	0.391	0.294	1						
TUG	0.060	0.006	0.330	0.492	−0.474	−0.442	−0.322	−0.294	−0.503	1					
Protein intake	−0.194	−0.220	−0.577	−0.169	0.299	0.173	0.074	0.079	0.266	−0.298	1				
Energy intake	0.231	0.230	−0.302	−0.237	0.1641	0.365	0.357	0.326	0.193	−0.294	0.758	1			
Exercise time	−0.123	−0.043	−0.229	−0.202	0.20	0.128	0.122 *	0.22	0.321	−0.167	0.258	0.262	1		
Strength time	−0.011	−0.016	−0.393	−0.342	0.302	0.251	0.211	0.273	0.304	−0.364	0.471	0.394	0.444	1	
Aerobics time	−0.010	−0.055	−0.292	−0.082	0.131	0.182	0.109	0.277	0.154	−0.227	0.314	0.284	0.219	0.281	1

Data presented as r_s_. Data analysed using Spearman’s rank unless otherwise stated. *, data analysed using Pearson’s correlation. FFMI, fat-free mass index; ASMMI, appendicular skeletal muscle mass index; FMI, fat mass index; 30STS, thirty second sit-to-stand; TUG, timed up-and-go.

**Table 7:** Multiple linear regression models of selected strength and function outcomes.

	Gait Speed (m∙s^−1^)		TUG (s)		Grip Strength (kg)		Shoulder Adduction Strength (kg)	
	β (95% CI)	*p*-Value	β (95% CI)	*p*-Value	β (95% CI)	*p*-Value	β (95% CI)	*p*-Value
Model 1 *n =* 121	*R*^2^ = 0.1327	**<0.001**	*R*^2^ = 0.1876	**<0.001**	*R*^2^ = 0.0106	0.496	*R*^2^ = 0.0510	0.1330
Protein (g∙kg^−1^∙day^−1^)	0.19 (0.05, 0.33)	**0.007**	−0.19 (−0.35, −0.02)	**0.025**	0.03 (−0.16, 0.22)	0.782	−0.96 (−6.65, 4.72)	0.738
Total exercise time (h∙week^−1^)	0.01 (0.00, 0.02)	0.194	−0.01 (−0.02, 0.00)	**0.034**	0.01 (−0.01, 0.02)	0.438	0.51 (−0.02, 1.05)	0.061
Model 2 *n =* 121	*R*^2^ = 0.1548	**<0.001**	*R*^2^ = 0.2654	**<0.001**	*R*^2^ = 0.5561	**<0.001**	*R*^2^ = 0.3918	**<0.001**
Protein (g∙kg^−1^∙day^−1^)	0.18 (0.04, 0.31)	**0.012**	−0.15 (−0.31, 0.00)	**0.049**	−0.02 (−0.15, 0.11)	0.739	−1.56 (−5.79, 2.67)	0.465
Total exercise time (h∙week^−1^)	0.01 (0.00, 0.01)	0.241	−0.01 (−0.02, 0.00)	0.066	0.00 (−0.01, 0.02)	0.661	0.45 (0.06, 0.85)	**0.025**
Age	−0.01 (−0.01, 0.00)	0.136	0.01 (0.00, 0.02)	**0.009**	−0.01 (−0.02, 0.00)	**0.002**	−0.08 (−0.32, 0.16)	0.504
Sex (male)	0.03 (−0.04, 0.11)	0.398	−0.07 (−0.14, 0.01)	0.092	0.45 (0.36, 0.55)	**<0.001**	10.11 (7.06, 13.16)	**<0.001**
Model 3 *n =* 121	*R*^2^ = 0.1558	**<0.001**	*R*^2^ = 0.2723	**<0.001**	*R*^2^ = 0.5884	**<0.001**	*R*^2^ = 0.4498	**<0.001**
Protein (g∙kg^−1^∙day^−1^)	0.17 (0.01, 0.33)	**0.038**	−0.11 (−0.29, 0.07)	0.234	0.07 (−0.05, 0.20)	0.256	2.02 (−2.46, 6.51)	0.373
Total exercise time (h∙week^−1^)	0.01 (0.00, 0.02)	0.229	−0.01 (−0.02, 0.00)	**0.045**	0.00 (−0.01, 0.01)	0.861	0.38 (0.01, 0.74)	**0.043**
Age	−0.01 (−0.01, 0.00)	0.131	0.01 (0.00, 0.02)	**0.010**	−0.01 (−0.02, 0.00)	**0.006**	−0.01 (−0.24, 0.22)	0.932
Sex (male)	0.05 (−0.11, 0.21)	0.529	−0.15 (−0.33, 0.03)	0.099	0.27 (0.12, 0.43)	**0.001**	3.30 (−2.00, 8.59)	0.220
Lean muscle mass (kg)	0.00 (−0.01, 0.01)	0.807	0.01 (0.00, 0.01)	0.234	0.01 (0.00, 0.02)	**0.004**	0.43 (0.15, 0.71)	**0.003**

Data presented as β coefficients with 95% confidence intervals. TUG, timed up-and-go. Bold indicates significant difference.

**Table 8:** Multiple linear regression model of selected body composition and bone outcomes.

	Spine BMD		Femur BMD		Lean Muscle (%)		Fat Mass (%)	
	β (95% CI)	*p*-Value	β (95% CI)	*p*-Value	β (95% CI)	*p*-Value	β (95% CI)	*p*-Value
Model 1 *n =* 121	*R*^2^ = 0.0740	**0.005**	*R*^2^ = 0.0366	0.082	*R*^2^ = 0.2789	**<0.001**	*R*^2^ = 0.2752	**<0.001**
Protein (g∙kg^−1^∙day^−1^)	−0.17 (−0.28, −0.06)	**0.004**	−0.09 (−0.17, −0.01)	**0.036**	0.08 (0.02, 0.13)	**0.005**	−0.08 (−0.14, −0.02)	**0.006**
Total exercise time (h∙week^−1^)	0.00 (−0.01, 0.01)	0.758	0.00 (−0.01, 0.01)	0.506	0.01 (0.00, 0.01)	**0.003**	−0.01 (−0.01, 0.00)	**0.004**
Model 2 *n =* 121	*R*^2^ = 0.3599	**<0.001**	*R*^2^ = 0.2004	**<0.001**	*R*^2^ = 0.6532	**<0.001**	*R*^2^ = 0.6542	**<0.001**
Protein (g∙kg^−1^∙day^−1^)	−0.17 (−0.26, 0.09)	**<0.001**	−0.10 (−0.18, −0.02)	**0.017**	0.08 (0.04, 0.11)	**<0.001**	−0.08 (−0.12, −0.04)	**<0.001**
Total exercise time (h∙week^−1^)	0.00 (−0.01, 0.01)	0.779	0.00 (−0.01, 0.01)	0.500	0.00 (0.00, 0.10)	**<0.001**	−0.01 (−0.01, 0.00)	**<0.001**
Age	0.00 (0.00, 0.01)	0.759	−0.00 (−0.01, 0.00)	0.575	0.00 (0.00, 0.00)	0.143	0.00 (0.00, 0.00)	0.147
Sex (male)	0.22 (0.17, 0.28)	**<0.001**	0.11 (0.07, 0.16)	**<0.001**	0.10 (0.08, 0.12)	**<0.001**	−0.11 (−0.13, −0.09)	**<0.001**

Data presented as β coefficients with 95% confidence intervals. BMD, bone mineral density. Bold indicates significant difference.

## Data Availability

The data presented in this study are available on request from the corresponding author. The data are not publicly available due to privacy and ethical reasons.

## Supplementary Materials

The following supporting information can be downloaded at: https://www.mdpi.com/article/10.3390/nu16091380/s1, Supplementary Figure S1. CONSORT Participant Flow Diagram; Supplementary Table S1. Body composition, strength and physical function in participants who meet functional criteria of sarcopenia versus high risk participants; Supplementary Table S2. Macronutrient dietary intake in participants who meet functional criteria of sarcopenia versus high risk participants; Supplementary Table S3. Physical activity levels in participants who meet functional criteria of sarcopenia versus high risk participants; Supplementary Table S4. Quality of life in participants who meet functional criteria of sarcopenia versus high risk participants; Supplementary Table S5. Quality of life SF-36 sub-domains and overall component scores by group.
