# Supplementary material for: Protein Intake and Physical Activity Levels as Determinants of Sarcopenia Risk in Community-Dwelling Older Adults
Source: Nutrients. 2024 May 2;16(9):1380. doi: 10.3390/nu16091380 (PMC11085115; doi:10.3390/nu16091380)
Supplement: Supplementary file 1 [file nutrients-16-01380-s001.zip › nutrients-2944139-supplementary.pdf]

**Supplementary Figure S1.** CONSORT Participant Flow Diagram

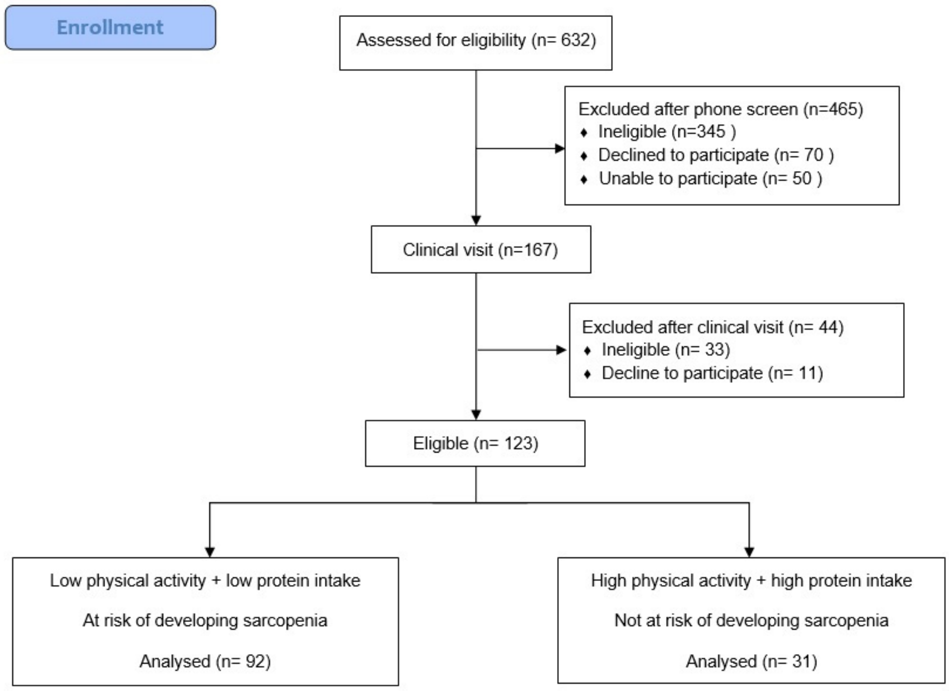

**Supplementary Table S1.** Body composition, strength and physical function in participants who meet functional criteria of sarcopenia versus high risk participants

| | **Functional Sarcopenia (n=17)** | **Other High Risk (n=75)** | p-values |
|---|---|---|---|
| Age | 72.2 (68.8-79.3) | 72.3 (68.6-75.3) | 0.530 |
| Female n (%) | 14 (82%) | 57 (80%) | 0.753 |
| | ***Body Composition*** | | |
| Weight (kg) | 80.7 (12.1) | 82.2 (14.9) | 0.699 |
| BMI ($kg{\cdot}m^2$) | 31.3 (5.1) | 30.2 (4.7) | 0.370 |
| FFM (kg) | 44.79 (42.33-46.88) | 44.43 (41.48-52.00) | 0.669 |
| FFMI ($kg{\cdot}m^2$) | 17.27 (16.46-18.83) | 16.77 (15.88-18.86) | 0.597 |
| Lean mass (%) | 50 (10) | 60 (10) | 0.350 |
| ASMM (kg) | 18.90 (17.23-19.88) | 18.43 (16.99-22.15) | 0.536 |
| ASMMI ($kg{\cdot}m^2$) | 7.12 (6.70-8.15) | 7.07 (6.46-8.03) | 0.829 |
| Fat mass (kg) | 37.18 (26.33-45.17) | 33.82 (27.65-41.07) | 0.721 |
| Fat mass index ($kg{\cdot}m^2$) | 15.12 (10.52-17.26) | 12.41 (10.37-15.23) | 0.347 |
| Fat (%)* | 50 (40-50) | 40 (40-50) | 0.241 |
| VAT mass (kg) | 1.12 (0.90-1.51) | 1.36 (0.99-1.93) | 0.263 |
| Total body bone mineral content (kg) | 2.10 (2.03-2.58) | 2.31 (2.07-2.77) | 0.170 |
| Spine BMD g/cm2 | 1.19 (0.24) | 1.21 (0.20) | 0.770 |
| Spine BMD T-scores, | 0.07 (1.95) | 0.16 (1.62) | 0.832 |
| Total hip BMD g/cm2 | 0.91 (0.19) | 0.95 (0.12) | 0.205 |
| Total hip BMD T-scores | -0.90 (1.52) | -0.55 (0.92) | 0.226 |
| | ***Strength*** | | |
| Five chair stand test (s) | *12.3 (10.0-17.2)* | *11.0 (9.4-13.2)* | *0.099* |
| Thirty second sit-to-stand (stands)* | 10.9 (2.7) | 13.1 (2.8) | **0.006** |
| Grip strength, total (kg) | 25.0 (20.0-28.0) | 26.0 (23.0-30.0) | 0.113 |
| Shoulder adduction strength (kg) | 8.0 ( 3.0-10.0) | 12.0 (7.5-16.5) | **0.002** |
| Shoulder abduction strength (kg) | 4.5 (3.0-7.5) | 7.5 (4.0-11.5) | **0.049** |
| | ***Physical Function*** | | |
| SPPB score (total) | 8.0 (8.0-11.0) | 11.0 (10.0-12.0) | **<0.001** |
| Gait speed ($m{\cdot}s^{-1}$) | 0.8 (0.8-0.8) | 1.0 (1.0-1.2) | **<0.001** |
| TUG (s) | 7.9 (7.0-11.0) | 7.0 (6.6-7.5) | **0.006** |

**Supplementary Table S2.** Macronutrient dietary intake in participants who meet functional criteria of sarcopenia versus high risk participants

| | **Functional Sarcopenia (n=17)** | **Other High Risk (n=75)** | ***p-values*** | ***Energy- adjusted p-values*** |
|---|---|---|---|---|
| ***Macronutrients*** | | | | |
| Energy (kJ·day$^{-1}$) | 5992 (1265) | 6257 (1477) | 0.506 | |
| Energy (kJ·kg$^{-1}$·day$^{-1}$) | 76.8 (17.2) | 77.6 (18.9) | 0.872 | |
| Protein (g·day$^{-1}$) | 65.1 (13.7) | 64.5 (12.7) | 0.867 | 0.321 |
| Protein (g·kg$^{-1}$·day$^{-1}$) | 0.8 (0.7-0.9) | 0.8 (0.7-0.9) | 0.552 | 0.238 |
| Carbohydrate (g·day$^{-1}$) | 138.9 (132.7-163.7) | 150.5 (121.3-184.4) | 0.359 | 0.460 |
| Fibre (g·day$^{-1}$) | 18.6 (17.5-27.7) | 19.7 (15.0-25.3) | 0.925 | 0.546 |
| Fat (g·day$^{-1}$)* | 62.5 (42.8-67.9) | 53.7 (44.1-69.1) | 0.942 | 0.564 |
| SFA (g·day$^{-1}$) | 22.0 (8.3) | 22.0 (8.4) | 0.989 | 0.571 |
| MUFA (g·day$^{-1}$) | 20.8 (14.5-27.9) | 20.2 (16.1-26.0) | 0.859 | 0.334 |
| PUFA (g·day$^{-1}$) | 7.5 (6.0-9.0) | 8.0 (6.4-10.2) | 0.307 | 0.410 |

**Supplementary Table S3.** Physical activity levels in participants who meet functional criteria of sarcopenia versus high risk participants

| | **Functional Sarcopenia (n=17)** | **Other High Risk (n=75)** | ***p-values*** |
|---|---|---|---|
| ***Activity Dimension Indices*** | | | |
| Vigorous Activity index (units·month$^{-1}$) | 0.0 (0.0-10.0) | 5.0 (0.0-15.0) | 0.555 |
| Leisure walking index (units·month$^{-1}$) | 8.0 (4.0-24.0) | 16.0 (8.0-24.0) | *0.099* |
| Moving index (units·month$^{-1}$) | 7.9 (3.0) | 8.6 (2.7) | 0.378 |
| Standing index (units·month$^{-1}$) | 4.0 (2.0-4.0) | 4.0 (2.0-4.0) | 0.854 |
| Sitting index (units·month$^{-1}$) | 2.4 (0.6) | 2.3 (0.8) | 0.875 |
| Total activity dimension indices | 33.2 (18.4) | 37.9 (15.4) | 0.278 |
| ***Activities*** | | | |
| Brisk walking (hours·week$^{-1}$) | 0.0 (0.0-0.3) | 0.0 (0.0-2.0) | 0.108 |
| Stretch/yoga/tai chi (hours·week$^{-1}$) | 0.0 (0.0-1.0) | 0.0 (0.0-1.0) | 0.845 |
| Aerobics (hours·week$^{-1}$) | 0.0 (0.0-0.0) | 0.0 (0.0-0.0) | 0.498 |
| Cycling (hours·week$^{-1}$) | 0.0 (0.0-0.0) | 0.0 (0.0-0.0) | 0.469 |
| Lap swimming (hours·week$^{-1}$) | 0.0 (0.0-0.0) | 0.0 (0.0-0.0) | 0.404 |
| Strength exercise (hours·week$^{-1}$) | 0.0 (0.0-0.0) | 0.0 (0.0-0.0) | 0.318 |
| Leisurely walking (hours·week$^{-1}$) | 0.5 (0.0-1.8) | 1.0 (0.0-2.0) | 0.550 |

**Supplementary Table S4.** Quality of life in participants who meet functional criteria of sarcopenia versus high risk participants

| | Functional Sarcopenia (n=17) | Other High Risk (n=75) | *p-values* |
|---|---|---|---|
| Physical function | 70 (40-80) | 85 (75-95) | **0.002** |
| Role limitations due to physical function | 75 (50-100) | 100 (75-100) | **0.035** |
| Pain | | | |
| General health | 72 (57-82) | 82 (67-87) | **0.046** |
| Energy | 60 (55-65) | 70 (60-80) | **0.005** |
| Social function | 88 (75-100) | 100 (88-100) | 0.011 |
| Role limitations due to emotional health | 100 (33-100) | 100 (100-100) | 0.255 |
| Emotional well-being | 80 (64-88) | 84 (76-88) | 0.242 |
| Physical component score | 44 (35-49) | 51 (47-55) | **0.004** |
| Mental component score | 53 (45-58) | 56 (52-58) | 0.222 |

**Supplementary Table S5.** Quality of life SF-36 sub-domains and overall component scores by group

| | High risk (*n*=92) | Low risk (*n*=31) | *p-values* |
|---|---|---|---|
| Physical function | 85 (70-95) | 95 (90-100) | **<0.001** |
| Role limitations due to physical function | 100 (75-100) | 100 (100-100) | **0.034** |
| Pain | 74 (62-100) | 74 (72-84) | 0.815 |
| General health | 81 (67-87) | 85 (77-92) | **0.006** |
| Energy | 68 (60-80) | 80 (70-85) | **0.011** |
| Social function | 100 (75-100) | 100 (88-100) | 0.469 |
| Role limitations due to emotional health | 100 (100-100) | 100 (100-100) | 0.054 |
| Emotional well-being | 84 (76-88) | 84 (80-92) | 0.240 |
| Physical component score | 50 (44-55) | 54 (50-57) | **0.013** |
| Mental component score | 55 (51-58) | 56 (53-58) | 0.378 |

Data presented as median (IQR).
